# Candidemia in Colombia: Species Distribution and Antifungal Susceptibility Profiles of Yeast Bloodstream Isolates from a Six-Year Multicentre Surveillance Study

**DOI:** 10.3390/jof12090640

**Published:** 2026-08-27

**Authors:** Andres Ceballos-Garzon, Nicolas Beltran, Valeri Saenz, Luz-Angela Pescador, Diego Josa, Alejandro Rodriguez-Bobadilla, Luisa-Fernanda Rodriguez, Rosalba Quintero-Munoz, Ivan-Felipe Gutierrez, Maria del Pilar Torres-Navarrete, Liliana Arevalo, Diana P. Cely-Ladino, Gloria Cortes, Luisa F. Ortiz-Torres, Rafael Pelufo, Nella Sanchez, Leidy Bogota, Claudia R. Sierra-Parada, Laura-Daniela Méndez, Maria J. Jimenez-A, Lizeth Paola Gutierrez, Jorge-Alberto Cortes, Aura-Lucia Leal

**Affiliations:** 1 Studies in Translational Microbiology and Emerging Diseases (MICROS) Research Group, School of Medicine and Health Sciences, Universidad del Rosario, Bogotá 111221, Colombia; 2 Faculty of Health Sciences, Universidad Colegio Mayor de Cundinamarca, Bogotá 110311, Colombia; 3 Departamento de Microbiología, Facultad de Medicina, Universidad Nacional de Colombia, Bogotá 111321, Colombia; 4 Grupo Para el Control de la Resistencia Bacteriana en Bogotá, GREBO, Bogotá 110911, Colombia; 5 Research Group Cardiovascular Medicine and Specialties of High Complexity, Fundación Clínica Shaio, Bogotá 110121, Colombia; 6 Hospital San Rafael de Facatativá, Facatativa 111071, Colombia; 7 Hospital Susana López Valencia, Popayan 190004, Colombia; 8 Clínica Infantil Colsubsidio, Bogotá 111221, Colombia; 9 Clínica Palermo, Bogotá 111311, Colombia; 10 Clínica La Colina, Bogotá 111156, Colombia; 11 Clínica del Country, Bogotá 111221, Colombia; 12 Hospital Universitario San Ignacio, Bogotá 110231, Colombia; 13 Fundación Hospital Pediátrico La Misericordia, Bogotá 110321, Colombia; 14 Hospital de Kennedy, Bogotá 110831, Colombia; 15 Hospital Tintal, Bogotá 11711, Colombia; 16 Hospital Universitario Santa Clara, Bogotá 111711, Colombia; 17 Hospital La Victoria, Bogotá 110411, Colombia; 18 Hospital San Blas, Bogotá 111711, Colombia; 19 Hospital Jorge Eliecer Gaitán, Bogotá 110231, Colombia; 20 Instituto Materno Infantil, Bogotá 111711, Colombia; 21 Hospital Rafael Uribe, Bogotá 111821, Colombia; 22 Fundación Santafé de Bogotá, Bogotá 110111, Colombia; 23 Clínica Infantil Santa María del Lago, Bogotá 111051, Colombia; 24 Clinica Reina Sofia, Bogotá 110111, Colombia; 25 Clínica Universitaria Colombia, Bogotá 111321, Colombia; 26 Fundacion Cardio Infantil, Bogotá 110111, Colombia; 27 Hospital Regional San Rafael de Cáqueza, Cundinamarca 250001, Colombia; 28 Hospital Universitario Nacional de Colombia, Bogotá 110911, Colombia

**Keywords:** candidemia, epidemiology, antifungal susceptibility, fluconazole resistance, multicentre study

## Abstract

A multicentre, laboratory-based analysis was conducted on aggregated data from 23 hospitals across Colombia. Of 1761 fungal isolates identified, 1642 were yeasts and constitute the focus of this study. Isolates were identified using the VITEK^®^2 system, with confirmatory identification by MALDI-TOF-MS for a subset. Antifungal susceptibility testing for fluconazole, voriconazole, amphotericin B, and the echinocandins was performed on *Candida albicans*, *C. parapsilosis*, and *C. tropicalis*. Data were stratified by clinical service: adult ICU, adult wards, paediatric ICU, neonatal ICU, and paediatric wards. Temporal trends were assessed using the Cochran–Armitage test. The most prevalent species were *C. albicans* (42%), *C. parapsilosis* (24%), *C. tropicalis* (11%), *C. glabrata* (7%), and *C. auris* (3%). *C. albicans* predominated in adult services, whereas *C. parapsilosis* was the leading pathogen across paediatric settings. Among species with validated breakpoints, the overall fluconazole resistance rate was 19%, driven primarily by *C. parapsilosis* (35%, exceeding 40% in some clinical services). Non-wild-type to voriconazole and amphotericin B was low (<5%), and echinocandin resistance was exceedingly rare. The substantial fluconazole resistance driven by *C. parapsilosis*, together with the emergence of *C. auris*, underscores the urgent need for sustained surveillance and species-directed antifungal stewardship programmes.

## 1. Introduction

Invasive fungal infections (IFIs) pose a persistent and major challenge to global health, associated with substantial morbidity, mortality, and economic burden [1,2]. The epidemiological landscape is dynamic, continuously shaped by evolving patient demographics, healthcare practices, antimicrobial pressure, and the emergence of resistant pathogens. A critical trend has been the shift in candidemia aetiology, with a relative decrease in *Candida albicans* and a concomitant rise in non-albicans Candida-related species (NAC), many exhibiting reduced susceptibility to first-line antifungals [3,4,5,6].

In Colombia, this challenge is particularly acute. The country reports one of the highest incidences of invasive candidiasis in Latin America (1.96 cases per 1000 admissions), significantly exceeding rates in regional and high-income settings [7,8,9]. This high disease burden converges with a hyperendemic status for dimorphic mycoses and the emergence of multidrug-resistant *Candidozyma* (formerly *Candida*) *auris*, creating a complex epidemiological scenario [10,11]. Despite a legislated universal healthcare system, significant disparities in diagnostic capacity and antifungal access persist between metropolitan and rural areas in Colombia, as recently documented [12]. Conventional mycological methods remain the diagnostic cornerstone, while access to advanced tools like MALDI-TOF MS and antifungal susceptibility testing (AFST) is limited and unevenly distributed [13].

Historical multicentre data are essential to navigate this epidemiological landscape. The early surveillance study by Cortés et al. (2001–2007) in Colombia established a baseline, reporting *C. albicans* as the predominant species (44.7%), followed by *C. parapsilosis* and *C. tropicalis* (each 13.6%), with a very low prevalence of *Nakaseomyces glabratus* (formerly *C. glabrata*) (1.7%) [14].

In the intervening years, the clinical and microbiological context has changed [15,16,17,18]. Widespread antiretroviral therapy may have altered the epidemiology of cryptococcosis. The potential spread of *C. auris*, increased use of antifungal prophylaxis and therapeutics, and changes in critical care necessitate an updated, multicentre assessment of bloodstream yeast epidemiology.

This study aimed to provide a comprehensive, multicentre analysis of the species distribution and in vitro antifungal susceptibility profiles of yeast isolates from bloodstream infections across a network of hospitals in Colombia during the six-year period from 2019 to 2024. Specifically, we sought to describe the current species prevalence, analyse adult-paediatric differences, determine contemporary resistance rates, and compare findings with historical national data to identify trends over nearly two decades.

## 2. Materials and Methods

### 2.1. Study Design and Setting

A multicentre, laboratory-based surveillance study was conducted using data aggregated from 23 hospitals within the surveillance network coordinated by the Grupo para el control de la Resistencia Bacteriana de Bogotá (GREBO). Participating institutions represent the highest tiers of the Colombian healthcare system, including fourth-level (high-complexity) and third-level (tertiary-care) referral centres. All hospitals contributed data as part of GREBO’s ongoing laboratory surveillance programme, which standardises the reporting of bloodstream infection isolates (https://www.grupogrebo.org/).

### 2.2. Isolate Collection and Identification

The study included all unique yeast isolates recovered from blood cultures between 1 January 2019 and 31 December 2024. Only the first isolate per patient was included in the analysis to avoid duplication and clustering bias. Subsequent isolates from the same patient were excluded.

Species identification was performed at each participating laboratory using the automated VITEK^®^ 2 system with the YST identification card (bioMérieux, Marcy-l’Étoile, France), following the manufacturer’s instructions. This methodology was consistent across all centres. For a convenience subset of isolates (28%), identification was confirmed by matrix-assisted laser desorption/ionisation time-of-flight mass spectrometry (MALDI-TOF MS) using the Bruker Biotyper system (Bruker Daltonics, Bremen, Germany). Protein extraction and spectral analysis were performed as previously described [15]. Presumptive *C. auris* isolates identified by VITEK^®^ 2 were confirmed by MALDI-TOF MS. In discordant cases (2%), the MALDI-TOF MS identification was considered definitive, and the isolate was reclassified accordingly.

### 2.3. Antifungal Susceptibility Testing

Antifungal susceptibility testing was performed as part of routine clinical practice at each participating institution and was conducted only for isolates of *C. albicans*, *C. parapsilosis*, and *C. tropicalis*, as these are the species for which validated CLSI clinical breakpoints are established. Testing was not systematically performed on all eligible isolates; rather, it was ordered at the discretion of the treating physician. Consequently, the number of isolates tested varies across species, antifungals, years, and clinical services. Testing was conducted using the VITEK^®^ 2 system with the AST-YS08 card (bioMérieux, Marcy-l’Étoile, France). This automated system employs a growth-based spectrophotometric method and has demonstrated high essential and categorical agreement compared to the CLSI M27 broth microdilution reference method for common *Candida* species [19,20]. The system provides standardized MICs aligned with CLSI methodologies and integrates updated species-specific clinical breakpoints (CBPs) from CLSI supplement M27M44S (3rd edition, 2022) to ensure consistent clinical reporting [21]. For amphotericin B, for which no CBPs are defined for *Candida* spp., MICs were interpreted using the epidemiological cut-off values (ECVs) provided in the CLSI document M57S (4th edition 2022) [22] to distinguish wild-type from non-wild-type isolates. MICs for 5-flucytosine are presented as a distribution, as no CBPs or ECVs are currently available for its interpretation against *Candida* species.

### 2.4. Data Management and Statistical Analysis of Temporal Trends

Data from each hospital’s laboratory information system were standardised and compiled monthly using WHONET software v5.6 (WHO Collaborating Centre for Surveillance of Antimicrobial Resistance). The consolidated database was rigorously reviewed for consistency and completeness. Data were stratified for analysis according to the patient’s location at the time of culture: Adult Intensive Care Unit (ICU), Adult Wards (non-ICU), Paediatric ICU, Neonatal ICU, and Paediatric Wards.

To assess whether the relative proportions of the main *Candida* species changed significantly over time, a chi-square test for linear trend (Cochran–Armitage test) was performed using GraphPad version 11. This analysis was conducted at two levels: globally, on the annual counts of each species against the total annual isolates across all services combined; and service-specifically, on the annual counts for each of the five clinical services (Adult ICU, Adult Ward, Paediatric ICU, Neonatal ICU, Paediatric Ward) individually. Given that multiple hypotheses were tested simultaneously, a Bonferroni correction was applied to control the family-wise error rate. The threshold was set at α = 0.05 divided by the number of tests performed within each analysis family; accordingly, a two-sided *p*-value < 0.01 was considered significant after correction.

### 2.5. Ethical Considerations

This study was based on the secondary analysis of fully anonymised, aggregated microbiological surveillance data generated as part of routine clinical practice and collected under the auspices of the GREBO network. According to national regulations (Resolución 8430 de 1993, Ministerio de Salud de Colombia) and international guidelines for public health surveillance of antimicrobial resistance, this type of study does not require review by a research ethics committee nor individual patient consent, as it poses no risk to patients and uses non-identifiable data.

## 3. Results

### 3.1. Overall Epidemiology and Species Distribution

**Figure 1:**
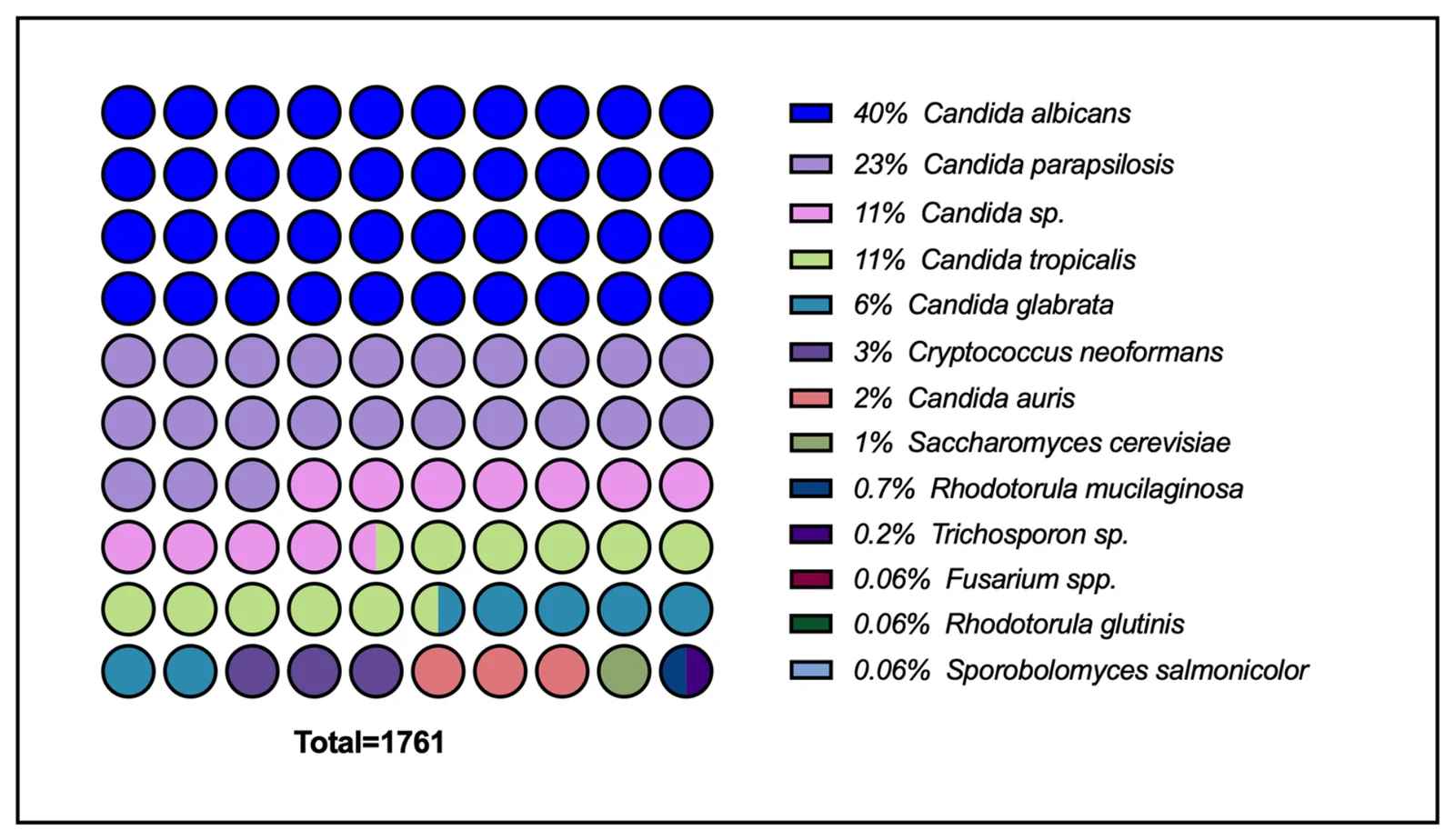
Distribution of fungal isolates recovered from bloodstream cultures across 23 Colombian hospitals (2019–2024).

Over the six-year study period (2019–2024), a total of 1761 fungal isolates were recovered from bloodstream cultures across the 23 participating hospitals (Figure 1). Of these, 1642 were yeasts, with members of the genus *Candida* and related genera (e.g., *Nakaseomyces*) predominating, constituting >93% of all yeast isolates. Other yeast genera identified at lower frequencies included *Cryptococcus*, *Rhodotorula*, and *Saccharomyces*. Among yeast species, *C. albicans* was the most prevalent, representing 42.3% (n = 694) of all yeast isolates. This was followed by *C. parapsilosis* (24.2%, n = 397), *C. tropicalis* (11.5%, n = 188), *C. glabrata* (6.7%, n = 110), and the emerging pathogen *C. auris* (3.1%, n = 51). Other *Candida* species and the remaining non-*Candida* yeasts collectively accounted for 12.3% (n = 202) of the total.

### 3.2. Distribution by Clinical Service

**Figure 2:**
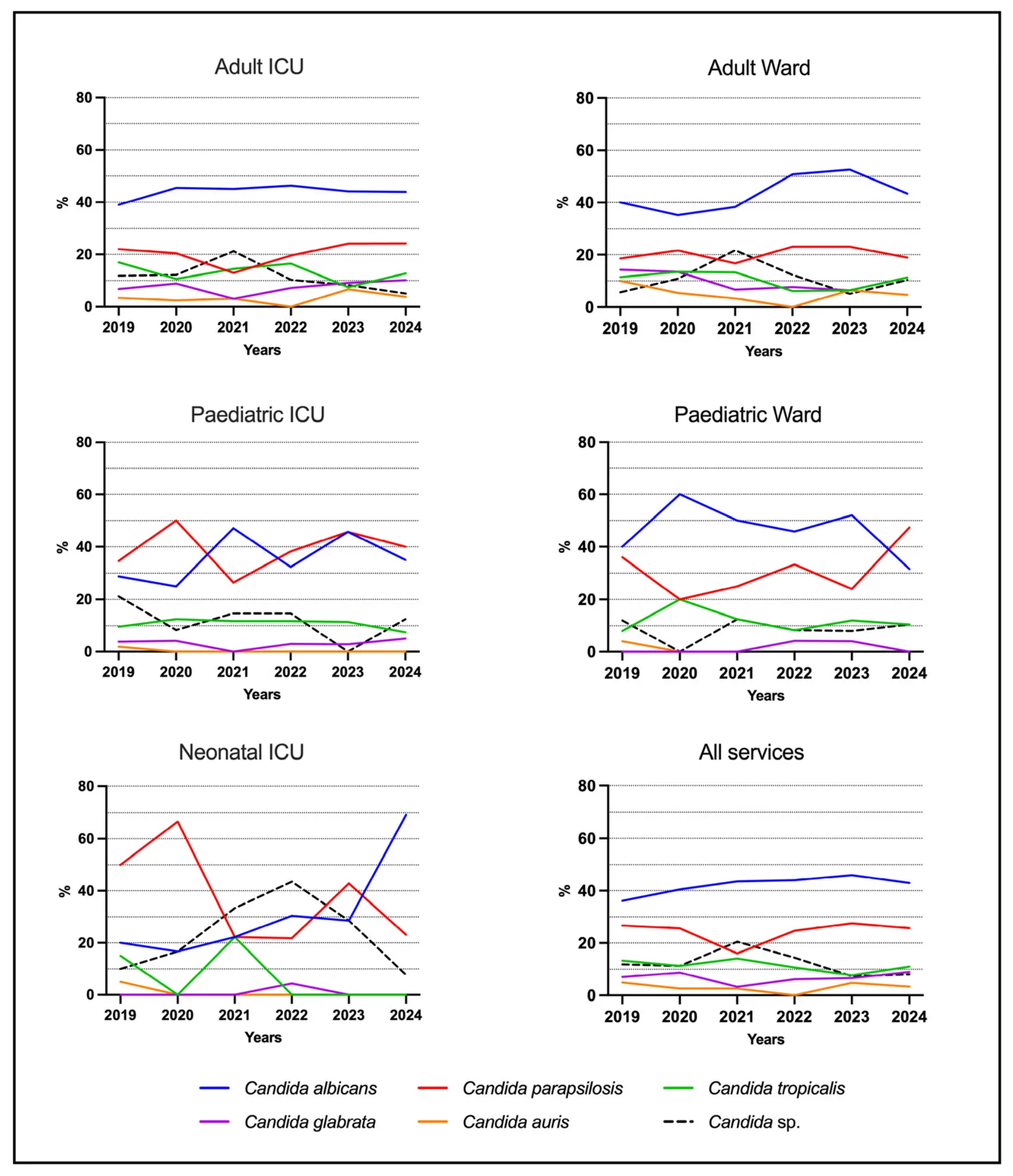
Temporal trends in species distribution among *Candida* bloodstream isolates, stratified by clinical service and year (2019–2024).

The distribution of the principal *Candida* species varied substantially between adult and paediatric services, with notable temporal fluctuations within some units, as detailed in Figure 2. In the adult ICU, from which 808 isolates were recovered, *C. albicans* was consistently the predominant species each year (44.1%, n = 356), followed by *C. parapsilosis* (20.0%, n = 162) and *C. tropicalis* (13.1%, n = 106). *C. auris* was identified in this setting, accounting for 3.3% (n = 27) of adult ICU isolates. A similar pattern of minimal annual variation was observed in adult hospital wards (non-ICU, 416 isolates), where *C. albicans* was the most frequent species (44.2%, n = 184), with *C. parapsilosis* consistently ranking second (20.2%, n = 84); *C. auris* was also detected, representing 5.0% (n = 21) of isolates. In contrast, the paediatric ICU (219 isolates) presented a distinct profile, with *C. parapsilosis* as the leading pathogen overall (38.4%, n = 84), surpassing *C. albicans* (34.7%, n = 76); a single isolate of *C. auris* was recorded in this unit. In the neonatal ICU (85 isolates), *C. parapsilosis* was the most common species overall (35.3%, n = 30), although a notable shift occurred in 2024, where *C.* *albicans* isolates outnumbered those of *C. parapsilosis*; one case of *C. auris* was also identified. In the paediatric wards (114 isolates), *C. albicans* was the most isolated species from 2019 to 2023; however, a marked increase in *C. parapsilosis* isolates in 2024 shifted the annual dominance to this species, resulting in a very close overall frequency between the two (*C. albicans* 44.7%, n = 51; *C. parapsilosis* 32.5%, n = 37). One case of *C. auris* was documented in this setting.

When aggregated, a clear epidemiological divergence was evident: in combined adult clinical settings (ICU and wards, n = 1224), *C. albicans* was the dominant pathogen (44.1%, n = 540), with *C. parapsilosis* second (20.1%, n = 246). Conversely, across all paediatric services (including ICUs and wards, n = 418), *C. parapsilosis* emerged as the most common species (38.0%, n = 159), followed by *C. albicans* (33.7%, n = 141). Analysing the overall annual trends for the entire cohort, the relative prevalence of the leading species remained stable throughout the six-year period. *C. albicans* consistently accounted for 36.1% to 46.0% of annual isolates, while *C. parapsilosis* represented 16.0% to 27.6% each year. The proportion of *C. tropicalis* fluctuated between 7.7% and 14.1%, and *C. glabrata* between 3.2% and 9.0%. Notably, *C. auris* was detected from 2019 onwards, with an annual prevalence ranging from 0% to 4.9%.

To formally evaluate these temporal trends, a Cochran–Armitage test for linear trends was performed with Bonferroni correction for multiple comparisons (adjusted α = 0.01). At the global level, no significant trend was detected for *C. albicans* (*p* = 0.32), *C. parapsilosis* (*p* = 0.18), *C. tropicalis* (*p* = 0.67), or *C. glabrata* (*p* = 0.09), confirming that the overall species distribution remained stable. At the service-specific level, none of the observed annual fluctuations within individual units reached statistical significance after correction (*p* > 0.01 for all comparisons). The low annual counts of *C. auris* precluded formal trend analysis for this emerging pathogen.

### 3.3. Antifungal Susceptibility Profiles

The analysis of antifungal resistance in adult settings revealed consistent species-specific patterns, though with varying magnitudes between the ICU and general wards (Table 1 and Table 2). Across both environments, *C. parapsilosis* exhibited the highest and most variable rates of fluconazole resistance, ranging from 25% to 80% annually, culminating in overall rates of 43.2% (n = 48/111) in the ICU and 40.4% (n = 19/47) in the general ward. In contrast, fluconazole resistance in *C. albicans* was substantially lower, particularly in the general ward (5.8%, n = 7/121) compared to the ICU (10.8%, n = 24/222), the latter influenced by a notable peak of 52.5% resistance in 2021. However, this single-year peak should be interpreted with caution as it reflects a specific temporal and service-related event and has not been confirmed by reference methods. *Candida tropicalis* showed intermediate fluconazole resistance (ICU: 24.2%; Ward: 14.8%). Resistance to voriconazole remained low (<5%) for all species in both settings, apart from *C. tropicalis* in the general ward (11.5%, n = 3/26). Resistance to echinocandins was a rare event, identified in single isolates of *C. albicans* (caspofungin, ICU) and *C. parapsilosis* (micafungin, ICU), and one *C. tropicalis* (caspofungin, general ward). Non-wild-type amphotericin B was uncommon and primarily associated with *C. parapsilosis* (ICU: 6.3%; Ward: 4.0%).

The resistance landscape in paediatric services (ICU, neonatal ICU, and general ward) (Table 3, Table 4 and Table 5) differed notably from that in adults, both in overall rates and species-specific trends. Fluconazole resistance remained a concern but was generally lower and more variable. *C. parapsilosis* continued to show the highest fluconazole resistance among paediatric isolates, with rates of 30.3% (n = 20/66) in the paediatric ICU, 14.3% (n = 3/21) in the neonatal ICU, and 23.5% (n = 8/34) in the general ward. In contrast, fluconazole resistance in *C. albicans* was markedly lower across all paediatric settings, not exceeding 2% in the paediatric ICU and general ward, and reaching 21.5% (n = 3/14) in the neonatal ICU. *C. tropicalis* exhibited highly variable but often elevated fluconazole resistance in paediatric cohorts, with peaks of 100% in small sample sets within the paediatric and neonatal ICUs. Resistance to voriconazole was low (<10%) across all species and settings. Notably, resistance to echinocandins was detected exclusively in *C. tropicalis* isolates from the paediatric ICU (caspofungin: 10%, n = 1/10; micafungin: 16.7%, n = 1/6). No non-wild-type amphotericin B was recorded in any paediatric isolate.

**Figure 3:**
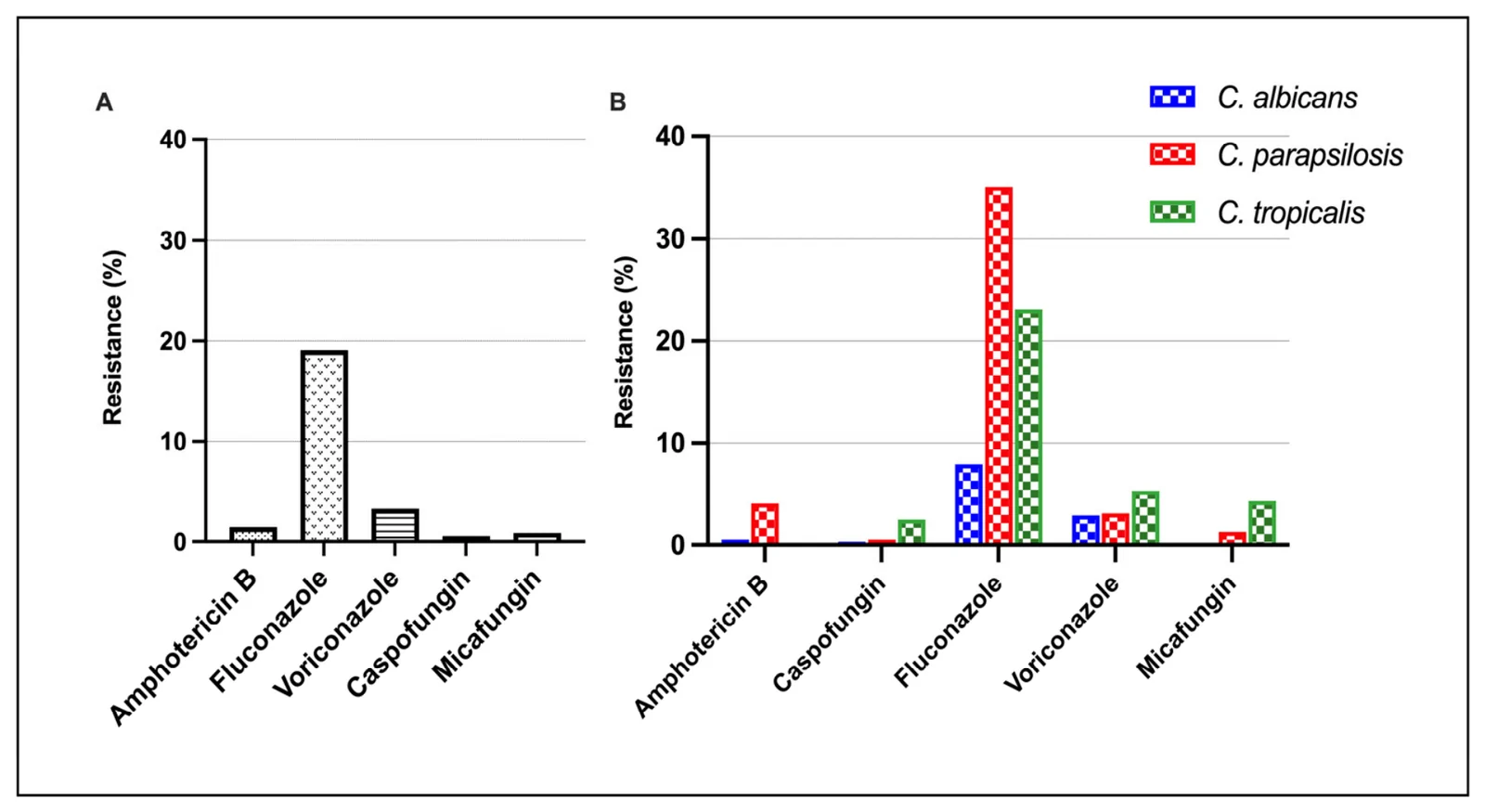
Overall antifungal resistance rates for *C. albicans*, *C. parapsilosis*, and *C. tropicalis* bloodstream isolates (2019–2024). (**A**) Global resistance/non-wild-type rates across all three species combined for each antifungal agent. (**B**) Species-specific resistance/non-wildtype rates to amphotericin B, caspofungin, fluconazole, voriconazole, and micafungin.

Aggregate antifungal resistance data for all tested isolates are presented in Figure 3. Among 839 isolates tested against fluconazole, the overall resistance rate was 19.1% (n = 160), driven primarily by the high resistance rate (35%) observed among *C. parapsilosis* isolates. Resistance to voriconazole was markedly lower, at 3.3% (26/786). Non-wild type to amphotericin B was rare, occurring in only 1.5% (6/393) of tested isolates. Resistance to the echinocandins caspofungin and micafungin was exceedingly uncommon, with rates of 0.6% (4/636) and 0.9% (2/232), respectively. Resistance percentages derived from small denominators should be interpreted with caution, as they are statistically unstable and may be misleading. The presentation of absolute numbers alongside percentages throughout this section allows readers to assess the reliability of each estimate and avoid overinterpretation.

## 4. Discussion

This multicentre surveillance study provides a six-year overview of the epidemiology of yeast bloodstream infections in several institutions in Colombia. Our findings reveal a dynamic epidemiological landscape, characterised by four key trends: a distinct divergence between adult and paediatric populations, the consolidation of *C. parapsilosis* as a pathogen of paramount importance in paediatrics, its associated high rates of antifungal resistance, and the concerning emergence of *C. auris.* Although derived from Colombia, these findings are likely generalisable to other Latin American and middle-income settings with comparable healthcare structures, and they offer a valuable benchmark for European surveillance systems, particularly given the high local incidence of candidemia and the recent documentation of azole-resistant *C. parapsilosis* and *C. auris*, threats that transcend geographical boundaries.

The species distribution observed in this study (2019–2024) marks a notable evolution from the historical baseline established by Cortés et al. in the previous decade (2001–2007) [14]. While *C. albicans* remains the most prevalent species overall (42.3% vs. 44.7% in 2001–2007), the ranking and proportion of NAC species have shifted significantly. *Candida parapsilosis* has risen from being the joint second most common species (13.6%) to a clear second place (24.2%), a trend that reflects its growing regional prominence and aligns with observations from other studies (e.g., Italy and Spain) [23,24,25,26]. Conversely, the proportion of *C. tropicalis* has decreased (from 13.6% to 11.5%). Perhaps the most striking new finding is the detection of *C. auris*, which accounted for 3.1% of isolates, a species absent in the earlier national surveillance. This aligns with its recent emergence as a global multidrug-resistant healthcare-associated pathogen [27].

A central finding is the stark difference in aetiology between adult and paediatric patients. In adult services, *C. albicans* predominated, consistent with global patterns in general and ICU populations [28]. In contrast, *C. parapsilosis* was the leading cause of fungemia across paediatric services. This is a well-documented phenomenon, in part attributed to the affinity of *C. parapsilosis* for biofilm formation on medical devices and its prevalence in the hands of healthcare workers, facilitating transmission in neonatal and paediatric intensive care settings [28,29,30]. Our data corroborate recent studies from Brazil, where *C. parapsilosis* was the predominant agent in neonatal candidemia [31]. The fluctuating annual dominance between *C. albicans* and *C. parapsilosis* observed within specific paediatric units underscores the dynamic nature of nosocomial transmission and possibly the impact of local infection prevention and control measures, warranting further unit-specific investigation. It is noteworthy that no significant temporal trends in species distribution were detected over the six-year study period, despite its overlap with the COVID-19 pandemic. In Colombia, antimicrobial stewardship programmes (ASPs, known locally as PROA) have been promoted since 2012 and became mandatory for all healthcare institutions with Resolution 2471 of 2022. All hospitals in our network had active ASPs throughout the study period, including antifungal stewardship components. Colombia entered the pandemic with pre-existing stewardship frameworks that were further strengthened through national initiatives. The absence of prolonged nationwide healthcare saturation may have enabled the sustained implementation of these measures, which may have contributed to the stability of species distribution and resistance patterns observed in our study.

The antifungal susceptibility data from this multicentre cohort reveal an overall fluconazole resistance rate of 19.1% which is substantially higher than the 6.8% reported in a multi-country Colombian–Ecuadorian–Venezuelan study two decades ago [32] and aligns more closely with the 18% resistance recently reported in a single-centre Colombian study investigating resistance mechanisms in human pathogenic yeast [11]. Crucially, this resistance is not uniformly distributed but is heavily concentrated in specific species. *C. parapsilosis* exhibited the highest rates of fluconazole resistance, reaching 43.2% and 40.5% in adult ICU and ward settings, respectively. This finding is consistent with reports from multiple countries across Europe, Africa, and Latin America [33,34]. Our findings are further supported by a recently published post-pandemic cohort study from a high-complexity teaching hospital in Cali, Colombia (2022–2023), which reported that non-albicans species predominated (61.5%) and documented an alarming fluconazole resistance rate of 28.6% in *C. parapsilosis* [35]. In contrast, a recent Colombian time-series analysis of invasive candidiasis reported a higher proportion of *C. albicans* (45–70%) and did not systematically report antifungal susceptibility data, which may reflect differences in case definition (all invasive candidiasis vs. bloodstream isolates only) and study design [36]. Nevertheless, both studies agree on the sustained predominance of *C. albicans* and the growing importance of *C. parapsilosis* in the Colombian context. The molecular basis for this has been partially elucidated in Colombia by Ceballos-Garzón et al. who identified Erg11p substitutions (i.e., Y132F, K143R, T220L) in fluconazole-resistant *C. parapsilosis* and *C. albicans* isolates [11]. In contrast, fluconazole resistance in paediatric *C. albicans* isolates was remarkably low (≤2%), a reassuring finding for a first-line agent still widely used in some paediatric settings. The low rates of resistance to voriconazole (<5% in most species/settings) and the exceedingly rare resistance to echinocandins (<1%) are encouraging and mirror susceptibility profiles reported in contemporary surveillance from Palestine and Turkey [37,38].

The sporadic but documented presence of *C. auris* calls for reinforced infection control protocols and active screening in high-risk adult units to prevent outbreaks, given its notorious capacity for transmission and multidrug resistance. Antifungal susceptibility testing for *C. auris* was not performed in routine practice across the network, as no CLSI or EUCAST clinical breakpoints (CBPs) are currently established for this species, precluding categorical interpretation of MIC results. However, provisional epidemiological cutoff values (ECVs) proposed by the CDC and CLSI/EUCAST offer a framework for distinguishing wild-type from non-wild-type isolates and could serve as an interim reference pending the formal validation of CBPs. Incorporating MIC testing against fluconazole, amphotericin B, and echinocandins for *C. auris* isolates, interpreted against these provisional ECVs, is therefore strongly encouraged in future surveillance cycles within this network.

This study has limitations inherent to its laboratory-based surveillance design. The lack of clinical data precludes analysis of patient outcomes, risk factors, or the impact of antifungal therapy. The variation in the number of isolates tested for AFST across species, years, and services reflects routine clinical practice rather than a standardized research protocol. This may introduce selection bias, as testing was not systematically performed for all eligible isolates. Furthermore, antifungal susceptibility testing was performed using the automated VITEK^®^ 2 system. While this reflects real-world practice in most Latin-American centres, as highlighted by recent capacity assessments [12,39,40], it is not a reference method and may present limitations [41]. However, the VITEK^®^ 2 AST-YS08 card has demonstrated high essential and categorical agreement with the CLSI reference method for common *Candida* species [19,20]. and our resistance rates are consistent with those from a prior single-centre study using the reference method [11]. In response to the high fluconazole resistance observed in *C. parapsilosis*, we have initiated an ongoing study to collect these isolates for confirmatory testing and molecular characterisation of resistance mechanisms. Regarding 5-flucytosine, the absence of validated CBPs or ECVs for *Candida* spp. complicates data interpretation. When applying the tentative ECVs proposed by Pfaller et al. [42] over 90% of the isolates would be categorised as non-wild type. This discordance underscores an urgent need for collaborative studies to establish robust, region-specific ECVs for 5-flucytosine in Latin American *Candida* populations and to validate the correlation between VITEK 2 results and reference methods for this agent. Despite these limitations, this multicentre analysis provides actionable data to support antifungal stewardship in Colombia.

## 5. Conclusions

This six-year multicentre surveillance study delineates the current and evolving landscape of yeast bloodstream infections in Colombia. It confirms the increase in non-*albicans Candida* species, with *C. parapsilosis* solidifying its position as the second most prevalent pathogen overall, a notable rise compared to previous national surveillance. This study confirms a distinct epidemiological profile in paediatric patients, dominated by *C. parapsilosis*, and highlights the significant challenge of fluconazole resistance particularly in adult settings. The detection of *C. auris* underscores the necessity for clinical laboratories to implement routine identification and antifungal susceptibility testing for this multidrug-resistant pathogen. Despite excellent retained susceptibility to echinocandins and amphotericin B, the findings argue strongly for the strengthening of antifungal stewardship programmes, guided by local epidemiology and rapid diagnostic identification. Ultimately, sustaining this surveillance is crucial to monitor trends, detect emerging resistance, and evaluate the impact of stewardship interventions, thereby improving outcomes for patients with invasive fungal infections in Colombia and similar settings.

## Figures and Tables

**Table 1:** Number of isolates tested (n) and resistance/non-wild-type rates (%) to amphotericin B, caspofungin, fluconazole, voriconazole, and micafungin for *Candida* bloodstream isolates in the Adult ICU service.

Adult ICU															
Microorganism	Antifungal Agent	2019		2020		2021		2022		2023		2024		Total	
		n	R (%)	n	R (%)	n	R (%)	n	R (%)	n	R (%)	n	R (%)	n	R (%)
***C. albicans***	Amphotericin B	26	0 (0)	29	0 (0)	31	0 (0)	16	0 (0)	8	0 (0)	19	0 (0)	129	0 (0)
	Caspofungin	20	0 (0)	29	0 (0)	12	0 (0)	25	0 (0)	26	0 (0)	48	1 (2.1)	160	1 (0.6)
	Fluconazole	33	1 (3)	36	1 (2.8)	40	21 (52.5)	27	0 (0)	31	1 (3.2)	55	0 (0)	222	24 (10.9)
	Voriconazole	30	0 (0)	35	1 (2.9)	36	5 (13.9)	27	0 (0)	31	1 (3.2)	55	1 (1.8)	214	8 (3.8)
	Micafungin	-	-	-	-	-	-	19	0 (0)	25	0 (0)	-	-	44	0 (0)
	Itraconazole	-	-	-	-	-	-	-	-	-	-	1	0 (0)	1	0 (0)
***C. tropicalis***	Amphotericin B	10	0 (0)	5	0 (0)	17	0 (0)	3	0 (0)	1	0 (0)	4	0 (0)	40	0 (0)
	Caspofungin	7	0 (0)	5	0 (0)	6	0 (0)	9	0 (0)	4	0 (0)	11	0 (0)	42	0 (0)
	Fluconazole	12	3 (25)	5	0 (0)	19	11 (58)	9	1 (11)	4	0 (0)	13	0 (0)	62	15 (24.2)
	Voriconazole	12	0 (0)	5	0 (0)	19	0 (0)	8	1 (12.5)	4	0 (0)	13	0 (0)	61	1 (1.6)
	Micafungin	-	-	-	-	-	-	5	0 (0)	4	0 (0)	-	-	9	0 (0)
***C. parapsilosis***	Amphotericin B	22	1 (4.5)	20	0 (0)	10	1 (10)	5	1 (20)	3	1 (33.3)	4	1 (25)	64	4 (6.25)
	Caspofungin	18	0 (0)	20	0 (0)	3	0 (0)	13	1 (7.7)	13	0 (0)	21	0 (0)	88	1 (1.15)
	Fluconazole	26	13 (50)	20	9 (45)	16	11 (69)	12	3 (25)	15	6 (40)	22	6 (27.3)	111	48 (43.25)
	Voriconazole	25	0 (0)	19	1 (5.3)	13	3 (23)	11	0 (0)	15	0 (0)	22	0 (0)	105	4 (3.8)
	Micafungin	-	-	-	-	-	-	9	1 (11)	13	0 (0)	-	-	22	1 (4.5)

n, number of MIC determinations per antifungal–species combination; since up to six antifungal agents were tested per isolate and not all agents were evaluated in every isolate, the cumulative total exceeds the number of unique isolates recovered from each clinical service. -, not tested.

**Table 2:** Number of isolates tested (n) and resistance/non-wild-type rates (%) to amphotericin B, caspofungin, fluconazole, voriconazole, and micafungin for *Candida* bloodstream isolates in the adult ward service.

Adult Ward															
Microorganism	Antifungal Agent	2019		2020		2021		2022		2023		2024		Total	
		n	R (%)	n	R (%)	n	R (%)	n	R (%)	n	R (%)	n	R (%)	n	R (%)
***C. albicans***	Amphotericin B	10	0 (0)	6	0 (0)	9	0 (0)	10	0 (0)	6	0 (0)	11	0 (0)	52	0 (0)
	Caspofungin	12	0 (0)	6	0 (0)	8	0 (0)	20	0 (0)	20	0 (0)	28	0 (0)	94	0 (0)
	Fluconazole	20	1 (5)	8	0 (0)	16	5 (31.3)	23	0 (0)	22	0 (0)	32	1 (3.1)	121	7 (5.8)
	Voriconazole	14	0 (0)	7	0 (0)	13	0 (0)	21	0 (0)	22	1 (4.5)	32	0 (0)	109	1 (0.9)
	Micafungin	-	-	-	-	2	0 (0)	15	0 (0)	19	0 (0)	-	-	36	0 (0)
	Itraconazole	-	-	-	-	-	-	-	-	-	-	1	0 (0)	1	0 (0)
***C. tropicalis***	Amphotericin B	3	0 (0)	2	0 (0)	4	0 (0)	3	0 (0)	1	0 (0)	3	0 (0)	16	0 (0)
	Caspofungin	1	0 (0)	2	0 (0)	3	0 (0)	2	0 (0)	1	0 (0)	8	1 (12.5)	17	1 (5.9)
	Fluconazole	5	2 (40)	2	0 (0)	7	2 (28.6)	3	0 (0)	1	0 (0)	9	0 (0)	27	4 (14.8)
	Voriconazole	4	1 (25)	2	0 (0)	7	1 (14.3)	3	0 (0)	1	0 (0)	9	0 (0)	26	3 (11.5)
	Micafungin	-	-	-	-	1	0 (0)	2	0 (0)	1	0 (0)	-	-	4	0 (0)
***C. parapsilosis***	Amphotericin B	7	0 (0)	4	0 (0)	4	0 (0)	5	1 (20)	3	0 (0)	2	0 (0)	25	1 (4)
	Caspofungin	10	0 (0)	4	0 (0)	4	0 (0)	7	0 (0)	7	0 (0)	9	0 (0)	41	0 (0)
	Fluconazole	13	5 (38.5)	5	0 (0)	5	4 (80)	7	3 (42.9)	8	4 (50)	9	3 (33.3)	47	19 (40.5)
	Voriconazole	7	0 (0)	5	0 (0)	5	0 (0)	7	0 (0)	7	1 (14.3)	9	0 (0)	40	1 (2.5)
	Micafungin	-	-	-	-	1	0 (0)	6	0 (0)	7	0 (0)	-	-	14	0 (0)

n, number of MIC determinations per antifungal–species combination; since up to six antifungal agents were tested per isolate and not all agents were evaluated in every isolate, the cumulative total exceeds the number of unique isolates recovered from each clinical service. -, not tested.

**Table 3:** Number of isolates tested (n) and resistance/non-wild-type rates (%) to amphotericin B, caspofungin, fluconazole, voriconazole, and micafungin for *Candida* bloodstream isolates in the paediatric ICU service.

Paediatric ICU															
Microorganism	Antifungal Agent	2019		2020		2021		2022		2023		2024		Total	
		n	R (%)	n	R (%)	n	R (%)	n	R (%)	n	R (%)	n	R (%)	n	R (%)
***C. albicans***	Amphotericin B	4	0 (0)	-	-	-	-	2	0 (0)	-	-	1	0 (0)	7	0 (0)
	Caspofungin	5	0 (0)	-	-	3	0 (0)	10	0 (0)	12	0 (0)	10	0 (0)	40	0 (0)
	Fluconazole	8	0 (0)	1	0 (0)	7	0 (0)	11	1 (9.1)	12	0 (0)	10	0 (0)	49	1 (2.0)
	Voriconazole	6	0 (0)	1	0 (0)	6	0 (0)	11	1 (9.1)	12	0 (0)	10	0 (0)	46	1 (2.2)
	Micafungin	-	-	-	-	4	0 (0)	9	0 (0)	12	0 (0)	-	-	25	0 (0)
***C. tropicalis***	Amphotericin B	2	0 (0)	-	-	2	0 (0)	-	-	-	-	-	-	4	0 (0)
	Caspofungin	3	0 (0)	-	-	-	-	4	0 (0)	2	1 (50)	1	0 (0)	10	1 (10)
	Fluconazole	4	1 (25)	-	-	2	100	4	0 (0)	2	0 (0)	2	0 (0)	14	3 (21.4)
	Voriconazole	4	0 (0)	-	-	2	50	4	0 (0)	2	0 (0)	2	0 (0)	14	1 (7.1)
	Micafungin	-	-	-	-	-	-	4	0 (0)	2	1 (50)	-	-	6	1 (16.7)
***C. parapsilosis***	Amphotericin B	8	0 (0)	4	0 (0)	-	-	-	-	1	0 (0)	-	-	13	0 (0)
	Caspofungin	6	0 (0)	7	0 (0)	2	0 (0)	10	0 (0)	12	0 (0)	12	0 (0)	49	0 (0)
	Fluconazole	15	2 (13.3)	8	2 (25)	5	0 (0)	10	5 (50)	13	5 (38.5)	15	6 (40)	66	20 (30.3)
	Voriconazole	14	0 (0)	5	1 (20)	5	0 (0)	9	0 (0)	12	0 (0)	15	2 (13.3)	60	3 (5.0)
	Micafungin	-	-	-	-	2	0 (0)	8	0 (0)	12	0 (0)	-	-	22	0 (0)

n, number of MIC determinations per antifungal–species combination; since up to six antifungal agents were tested per isolate and not all agents were evaluated in every isolate, the cumulative total exceeds the number of unique isolates recovered from each clinical service. -, not tested.

**Table 4:** Number of isolates tested (n) and resistance/non-wild-type rates (%) to amphotericin B, caspofungin, fluconazole, voriconazole, and micafungin for *Candida* bloodstream isolates in the neonatal ICU service.

Neonatal ICU															
Microorganism	Antifungal Agent	2019		2020		2021		2022		2023		2024		Total	
		n	R (%)	n	R (%)	n	R (%)	n	R (%)	n	R (%)	n	R (%)	n	R (%)
***C. albicans***	Amphotericin B	2	0 (0)	-	-	2	0 (0)	2	0 (0)	2	0 (0)	1	0 (0)	9	0 (0)
	Caspofungin	2	0 (0)	-	-	2	0 (0)	1	0 (0)	1	0 (0)	3	0 (0)	9	0 (0)
	Fluconazole	3	1 (33.3)	-	-	3	1 (33.3)	2	1 (50)	2	0 (0)	4	0 (0)	14	3 (21.5)
	Voriconazole	3	0 (0)	-	-	3	0 (0)	2	1 (50)	2	0 (0)	4	0 (0)	14	1 (7.1)
	Micafungin	-	-	-	-	2	0 (0)	1	0 (0)	1	0 (0)	-	-	4	0 (0)
***C. tropicalis***	Amphotericin B	2	0 (0)	-	-	1	0 (0)	-	-	-	-	-	-	3	0 (0)
	Caspofungin	1	0 (0)	-	-			-	-	-	-	-	-	1	0 (0)
	Fluconazole	2	1 (50)	-	-	1	1 (100)	-	-	-	-	-	-	3	2 (66.7)
	Voriconazole	2	0 (0)	-	-	1	0 (0)	-	-	-	-	-	-	3	0 (0)
	Micafungin	-	-	-	-	-	-	-	-	-	-	-	-		
***C. parapsilosis***	Amphotericin B	6	0 (0)	1	0 (0)	-	-	3	0 (0)	3	0 (0)			13	0 (0)
	Caspofungin	5	0 (0)	1	0 (0)	2	0 (0)	5	0 (0)	1	0 (0)	3	0 (0)	17	0 (0)
	Fluconazole	6	2 (33.3)	2	0 (0)	2	0 (0)	5	1 (20)	3	0 (0)	3	0 (0)	21	3 (14.3)
	Voriconazole	6	0 (0)	2	0 (0)	2	0 (0)	4	0 (0)	3	0 (0)	2	0 (0)	19	0 (0)
	Micafungin	-	-	-	-	2	0 (0)	5	0 (0)	1	0 (0)	-	-	8	0 (0)

n, number of MIC determinations per antifungal–species combination; since up to six antifungal agents were tested per isolate and not all agents were evaluated in every isolate, the cumulative total exceeds the number of unique isolates recovered from each clinical service. -, not tested.

**Table 5:** Number of isolates tested (n) and resistance/non-wild-type rates (%) to amphotericin B, caspofungin, fluconazole, voriconazole, and micafungin for *Candida* bloodstream isolates in the paediatric ward service.

Paediatric Ward															
Microorganism	Antifungal Agent	2019		2020		2021		2022		2023		2024		Total	
		n	R (%)	n	R (%)	n	R (%)	n	R (%)	n	R (%)	n	R (%)	n	R (%)
***C. albicans***	Amphotericin B	3	0 (0)	1	0 (0)	1	0 (0)					1	0 (0)	6	0 (0)
	Caspofungin	6	0 (0)	1	0 (0)	4	0 (0)	11	0 (0)	8	0 (0)	3	0 (0)	33	0 (0)
	Fluconazole	8	0 (0)	1	0 (0)	5	0 (0)	11	0 (0)	8	0 (0)	4	0 (0)	37	0 (0)
	Voriconazole	7	1 (14.3)	1	0 (0)	4	0 (0)	10	0 (0)	8	0 (0)	4	0 (0)	34	1 (3)
	Micafungin	-	-	-	-	3	0 (0)	10	0 (0)	8	0 (0)	-	-	21	0 (0)
***C. tropicalis***	Amphotericin B	2	0 (0)	-	-	2	0 (0)	-	-	-	-	-	-	4	0 (0)
	Caspofungin	2	0 (0)	1	0 (0)			2	0 (0)	2	0 (0)	2	0 (0)	9	0 (0)
	Fluconazole	2	0 (0)	1	0 (0)	2	2 (100)	2	0 (0)	2	1 (50)	2	0 (0)	11	3 (27.3)
	Voriconazole	2	0 (0)	-	-	2	0 (0)	2	0 (0)	2	1 (50)	2	0 (0)	10	1 (10.0)
	Micafungin	-	-	-	-	-	-	2	0 (0)	2	0 (0)	-	-	4	0 (0)
***C. parapsilosis***	Amphotericin B	6	0 (0)	-	-	1	0 (0)	-	-	1	0 (0)	-	-	8	0 (0)
	Caspofungin	5	0 (0)	-	-	1	0 (0)	8	0 (0)	4	0 (0)	8	0 (0)	26	0 (0)
	Fluconazole	9	1 (11.1)	-	-	3	2 (66.6)	8	0 (0)	5	3 (60)	9	2 (22.2)	34	8 (23.5)
	Voriconazole	9	0 (0)	-	-	3	0 (0)	7	0 (0)	5	0 (0)	7	0 (0)	31	0 (0)
	Micafungin	-	-	-	-	1	0 (0)	8	0 (0)	4	0 (0)	-	-	13	0 (0)

n, number of MIC determinations per antifungal–species combination; since up to six antifungal agents were tested per isolate and not all agents were evaluated in every isolate, the cumulative total exceeds the number of unique isolates recovered from each clinical service. -, not tested.

## Data Availability

All relevant data are presented within the manuscript. Additional information may be requested from the corresponding author.

## Abbreviations

The following abbreviations are used in this manuscript:

AFST	Antifungal susceptibility testing
CBP	Clinical breakpoint
CLSI	Clinical and Laboratory Standards Institute
ECV	Epidemiological cut-off value
GREBO	Grupo para el Control de la Resistencia Bacteriana de Bogotá
ICU	Intensive care unit
MALDI-TOF MS	Matrix-assisted laser desorption/ionisation time-of-flight mass spectrometry
MIC	Minimum inhibitory concentration

## References

[1] Denning D.W. (2024). Global Incidence and Mortality of Severe Fungal Disease. Lancet Infect. Dis..

[2] Bongomin F., Gago S., Oladele R.O., Denning D.W. (2017). Global and Multi-National Prevalence of Fungal Diseases—Estimate Precision. J. Fungi.

[3] Ricotta E.E., Lai Y.L., Babiker A., Strich J.R., Kadri S.S., Lionakis M.S., Prevots D.R., Adjemian J. (2020). Invasive Candidiasis Species Distribution and Trends, United States, 2009–2017. J. Infect. Dis..

[4] Wiederhold N.P. (2017). Antifungal Resistance: Current Trends and Future Strategies to Combat. Infect. Drug Resist..

[5] Guinea J. (2014). Global Trends in the Distribution of *Candida* Species Causing Candidemia. Clin. Microbiol. Infect..

[6] Gold J.A.W., Benedict K., Lyman M., Toda M., Little J.S., Ostrosky-Zeichner L. (2026). *Candida glabrata* Emerges as the Most Common Cause of Candidemia: Analysis of a Large Hospital-Based Database, United States, 2016–2024. Clin. Infect. Dis..

[7] Cortés J.A., Ruiz J.F., Melgarejo-Moreno L.N., Lemos E.V. (2020). Candidemia in Colombia. Biomedica.

[8] Barahona-Correa J.E., Calvo-Valderrama M.G., Romero-Alvernia D.M., Angulo-Mora J., Alarcon-Figueroa L.F., Rodriguez-Malagon M.N., Garzon-Herazo J.R. (2019). Epidemiology of Candidemia at a University Hospital in Colombia, 2008-2014. Univ. Med..

[9] Colombo A.L., Tobón A., Restrepo A., Queiroz-Telles F., Nucci M. (2011). Epidemiology of Endemic Systemic Fungal Infections in Latin America. Med. Mycol..

[10] Giusiano G., Gómez B.L. (2025). Trends in the Epidemiology of Systemic Endemic Mycoses in Latin America. Med. Mycol..

[11] Ceballos-Garzon A., Peñuela A., Valderrama-Beltrán S., Vargas-Casanova Y., Ariza B., Parra-Giraldo C.M. (2023). Emergence and Circulation of Azole-Resistant C. Albicans, C. Auris and C. Parapsilosis Bloodstream Isolates Carrying Y132F, K143R or T220L Erg11p Substitutions in Colombia. Front. Cell. Infect. Microbiol..

[12] Ceballos-Garzon A., Escandon P., Micelly J., Cornely O.A., Berrio I., Salmanton-García J. (2026). Diagnostic and Therapeutic Capacity for Fungal Infections in Colombia: An ESCMID-EFISG Nationwide Multicenter Assessment of Mycological Healthcare. Int. J. Infect. Dis..

[13] Pasqualotto A.C., Berrio I., Ortiz B., Maquera-Afaray J., Fernández N., Varela D., Cuéllar L.E., Pérez F., Perurena Lancha M.R., Perales D. (2026). Fungal Diagnostics and Antifungal Drug Access in Latin America and the Caribbean: An ESCMID EFISG Multinational Survey. Nat. Commun..

[14] Cortés J.A., Reyes P., Gómez C., Buitrago G., Leal A.L. (2011). Fungal Bloodstream Infections in Tertiary Care Hospitals in Colombia. Rev. Iberoam. Micol..

[15] Ceballos-Garzón A., Cortes G., Morio F., Zamora-Cruz E.L., Linares M.Y., Ariza B.E., Valderrama S.L., Garzón J.R., Alvarez-Moreno C.A., Le Pape P. (2019). Comparison between MALDI-TOF MS and MicroScan in the Identification of Emerging and Multidrug Resistant Yeasts in a Fourth-Level Hospital in Bogotá, Colombia. BMC Microbiol..

[16] Parra-Giraldo C.M., Valderrama S.L., Cortes-Fraile G., Garzón J.R., Ariza B.E., Morio F., Linares-Linares M.Y., Ceballos-Garzón A., de la Hoz A., Hernandez C. (2018). First Report of Sporadic Cases of *Candida auris* in Colombia. Int. J. Infect. Dis..

[17] Motoa G., Muñoz J.S., Oñate J., Pallares C.J., Hernández C., Villegas M.V. (2017). Epidemiology of *Candida* Isolates from Intensive Care Units in Colombia from 2010 to 2013. Rev. Iberoam. Micol..

[18] Maldonado N.A., Cano L.E., De Bedout C., Arbeláez C.A., Roncancio G., María Tabares A., Robledo C.G., Robledo J., Grupo Germen (2014). Association of Clinical and Demographic Factors in Invasive Candidiasis Caused by Fluconazole-Resistant *Candida* Species: A Study in 15 Hospitals, Medellín, Colombia 2010–2011. Diagn. Microbiol. Infect. Dis..

[19] Alfouzan W., Al-Enezi T., AlRoomi E., Sandhya V., Chandy R., Khan Z.U. (2017). Comparison of the VITEK 2 Antifungal Susceptibility System with Etest Using Clinical Isolates of *Candida* Species. Rev. Iberoam. Micol..

[20] Lee H., Choi S.H., Oh J., Koo J., Lee H.J., Cho S., Shin J.H., Lee H.K., Kim S., Lee C.H. (2022). Comparison of Six Antifungal Susceptibilities of 11 *Candida* Species Using the VITEK2 AST–YS08 Card and Broth Microdilution Method. Microbiol. Spectr..

[21] CLSI (2026). Performance Standards for Antifungal Susceptibility Testing of Yeast.

[22] CLSI (2022). CLSI Supplement M57S Epidemiological Cutoff Value for Antifungal Susceptibility Testing.

[23] Franconi I., Rizzato C., Tavanti A., Falcone M., Lupetti A. (2023). Paradigm Shift: *Candida parapsilosis* Sensu Stricto as the Most Prevalent *Candida* Species Isolated from Bloodstream Infections with Increasing Azole-Non-Susceptibility Rates: Trends from 2015–2022 Survey. J. Fungi.

[24] Bassetti M., Merelli M., Righi E., Diaz-Martin A., Rosello E.M., Luzzati R., Parra A., Trecarichi E.M., Sanguinetti M., Posteraro B. (2013). Epidemiology, Species Distribution, Antifungal Susceptibility, and Outcome of Candidemia across Five Sites in Italy and Spain. J. Clin. Microbiol..

[25] Escribano P., Guinea J. (2022). Fluconazole-Resistant *Candida* Parapsilosis: A New Emerging Threat in the Fungi Arena. Front. Fungal Biol..

[26] Mesini A., Mikulska M., Giacobbe D.R., Del Puente F., Gandolfo N., Codda G., Orsi A., Tassinari F., Beltramini S., Marchese A. (2020). Changing Epidemiology of Candidaemia: Increase in Fluconazole-Resistant Candida Parapsilosis. Mycoses.

[27] Lockhart S.R., Etienne K.A., Vallabhaneni S., Farooqi J., Chowdhary A., Govender N.P., Colombo A.L., Calvo B., Cuomo C.A., Desjardins C.A. (2017). Simultaneous Emergence of Multidrug-Resistant *Candida auris* on 3 Continents Confirmed by Whole-Genome Sequencing and Epidemiological Analyses. Clin. Infect. Dis..

[28] Vazquez J.A., Whitaker L., Zubovskaia A. (2025). Invasive Candidiasis in the Intensive Care Unit: Where Are We Now?. J. Fungi.

[29] Asadzadeh M., Ahmad S., Al-Sweih N., Hagen F., Meis J.F., Khan Z. (2019). High-Resolution Fingerprinting of *Candida parapsilosis* Isolates Suggests Persistence and Transmission of Infections among Neonatal Intensive Care Unit Patients in Kuwait. Sci. Rep..

[30] Hare R.K., Arastehfar A., Rosendahl S., Charsizadeh A., Daneshnia F., Eshaghi H., Mirhendi H., Boekhout T., Hagen F., Arendrup M.C. (2022). Candidemia among Hospitalized Pediatric Patients Caused by Several Clonal Lineages of *Candida parapsilosis*. J. Fungi.

[31] da Silva C.M., de Carvalho A.M.R., Macêdo D.P.C., Jucá M.B., Amorim R.d.J.M., Neves R.P. (2023). Candidemia in Brazilian Neonatal Intensive Care Units: Risk Factors, Epidemiology, and Antifungal Resistance. Braz. J. Microbiol..

[32] De Bedout C., Ayabaca J., Vega R., Méndez M., Santiago A.R., Pabón M.L., Tabares A., Arango M., Restrepo A., Newell V. (2003). Evaluación de La Susceptibilidad de Especies de Candida al Fluconazol Por El Método de Difusión de Disco. Biomedica.

[33] Ünal N., Spruijtenburg B., Arastehfar A., Gümral R., de Groot T., Meijer E.F.J., Türk-Dağı H., Birinci A., Hilmioğlu-Polat S., Meis J.F. (2024). Multicentre Study of *Candida parapsilosis* Blood Isolates in Türkiye Highlights an Increasing Rate of Fluconazole Resistance and Emergence of Echinocandin and Multidrug Resistance. Mycoses.

[34] Daneshnia F., de Almeida Júnior J.N., Ilkit M., Lombardi L., Perry A.M., Gao M., Nobile C.J., Egger M., Perlin D.S., Zhai B. (2023). Worldwide Emergence of Fluconazole-Resistant Candida Parapsilosis: Current Framework and Future Research Roadmap. Lancet Microbe.

[35] Muñoz-Lombo J.P., Cardales-Arizal W.D., Vallejo-Serna R.A., Berrio I. (2026). Shift in Candidemia Epidemiology and Emerging Fluconazole Resistance in *Candida parapsilosis*: A Post-Pandemic Cohort Study in a Colombian High-Complexity Teaching Hospital. J. Fungi.

[36] Oñate-Gutiérrez J.M., Alvarez-Moreno C.A., Cañadas-Aragón C., Vergara-Samur H. (2026). Temporal Trends and Seasonality of Invasive Candidiasis During and After the COVID-19 Pandemic: An Interrupted Time Series Analysis in Colombia. J. Fungi.

[37] Baniodeh H., Abu-Helu R., Abulihya M., Awwad M.Y., Dawoud A., Tebbji F., Sellam A. (2024). The First Prevalence and Antifungal Susceptibility Profile of *Candida* Infections in Palestine, 2022. BMC Infect. Dis..

[38] Dalyan Cilo B. (2023). Species Distribution and Antifungal Susceptibilities of *Candida* Species Isolated From Blood Culture. Cureus.

[39] Maquera-Afaray J., Cuéllar L.E., Cornely O.A., Salmanton-García J., IFI Diagnostic and Treatment Capacity Collaborator Team Peru (2025). Fungi under Fire: Diagnostic Capacities and Antifungal Availability in Peruvian Healthcare Facilities. Microbiol. Spectr..

[40] Riera F., Caeiro J.P., Cornely O.A., Salmanton-García J., Argentinian IFI Diagnostic and Treatment Capacity Group (2023). The Argentinian Landscape of Mycological Diagnostic Capacity and Treatment Accessibility. Med. Mycol..

[41] Ceballos-Garzon A., Garcia-Effron G., Cordoba S., Rodriguez J.Y., Alvarez-Moreno C., Le Pape P., Parra-Giraldo C.M., Morales-López S. (2022). Head-to-head Comparison of CLSI, EUCAST, Etest and VITEK®2 Results for *Candida auris* Susceptibility Testing. Int. J. Antimicrob. Agents.

[42] Pfaller M.A., Espinel-Ingroff A., Canton E., Castanheira M., Cuenca-Estrella M., Diekema D.J., Fothergill A., Fuller J., Ghannoum M., Jones R.N. (2012). Wild-Type MIC Distributions and Epidemiological Cutoff Values for Amphotericin B, Flucytosine, and Itraconazole and *Candida* Spp. as Determined by CLSI Broth Microdilution. J. Clin. Microbiol..

